# The use of sialic acids as attachment factors is a common feature of *Enterovirus*-D species

**DOI:** 10.1128/jvi.00429-25

**Published:** 2025-05-13

**Authors:** Typhaine Filhol, Alice Mac Kain, Marie-Line Joffret, Nolwenn Jouvenet, Vincent Caval, Maël Bessaud

**Affiliations:** 1 Virus Sensing and Signaling Unit, Department of Virology, Institut Pasteur, Université Paris Cité, CNRS UMR 3569 555089, Paris, France; 2 Laboratoire associé au Centre national de référence entérovirus/paréchovirus, Institut Pasteur 27058, Paris, France; University of Kentucky College of Medicine, Lexington, Kentucky, USA

**Keywords:** enterovirus, virus entry, zoonotic infections, receptors, enterovirus D, EV-D111, *Enterovirus deconjuncti*

## Abstract

Among the hundreds of enteroviruses (EVs) infecting humans, the members of the species EV-D (*Enterovirus deconjuncti*) display original traits. First, only five serotypes are known within this species, while other EV species have tens of serotypes each. Second, EV-Ds display a wide variety of tropisms: EV-D68s are respiratory viruses, EV-D70s have an ocular tropism, while EV-D94s, EV-D111s, and EV-D120s seem to be enteric viruses. Besides, while EV-D68s, EV-D70s, and EV-D94s have been detected in humans, EV-D120s were found exclusively in non-human primates, and the last virus type, EV-D111, was found in both. This and other observations have led to the hypothesis that EV-Ds could have a zoonotic origin. Previous studies have shown that EV-D68, EV-D70, and EV-D94 use sialic acids (Sias) as cellular attachment factors. We investigated the role of Sias in EV-D111 infection using sialidase treatments and loss-of-function experiments in human and simian cells. Assessing viral RNA yield by RT-qPCR analyses and infectious viral particle production by titration assays showed that the absence of Sias at the cell surface significantly slowed down EV-D111 infection kinetics without abolishing it. This suggests that Sia acts as an attachment factor. While EVs generally do not use Sias, EV-Ds seem to rely on them for optimal replication in cultured cells. Sia usage may therefore be an ancestral trait of this species. We also studied EV-B114, a simian enterovirus, and found that it does not use Sias. Our work provides new insight regarding an enterovirus that circulates in humans and exhibits unusual ecological traits.

**IMPORTANCE:**

Except for a few epidemics in the 1970s and 1980s, the impact of EV-Ds on human health remained modest until the 2010s. In 2014, EV-D68 was occasionally responsible for severe respiratory distress and fatal cases of muscular paralysis. EV-Ds have thus the ability to become pathogenic in humans, hence the importance of studying them. The recently discovered EV-D111, of which only a few isolates are available, has been detected in both human and simian samples, suggesting a potential zoonotic origin. We characterized the early steps of EV-D111 replication, with a focus on its ability to use Sias as attachment factors. We found that EV-D111, like other members of the EV-D species, but unlike most EVs, relies on Sia for optimal replication. Our work provides a better understanding of EV-D111 biology, which is essential to determine its tropism and its potential to emerge in humans.

## INTRODUCTION

The *Picornaviridae* family includes naked viruses (30 nm in diameter) with an icosahedral capsid and a genome consisting of a single-stranded RNA molecule of positive polarity. Viruses belonging to this genus, whose generic name is “enterovirus” (EV), have a genome of around 7,400 nucleotides (1). It contains a long open reading frame encoding a polyprotein that is subsequently cleaved into several functional proteins by viral proteases (2, 3). Recently, an additional short open reading frame has been discovered in some EVs (4, 5). The *Enterovirus* genus currently comprises 15 species (6, 7). Seven of these species contain viruses that infect humans. For a long time, human EVs were classified based on phenotypic criteria, and in particular according to their tropism. Respiratory viruses were referred to as “rhinoviruses” while enteric viruses were referred to as coxsackieviruses A and B or as echoviruses, depending on their pathogenicity in mice. This classification method was abandoned in the 1960s and 1970s. Since then, newly identified enterovirus serotypes have simply been assigned a number as they are discovered. The current classification is now based on phylogenetic criteria and molecular typing methods. It classifies the EVs into species and virus types, regardless of the phenotypic properties of the viruses. Consequently, the respiratory virus types and the enteric virus types are no longer separated into specific species. The EV species were recently renamed with a binomial nomenclature. RV-A (*Enterovirus alpharhino*), RV-B (*E. betarhino*), and RV-C (*E. cerhino*) consist exclusively of respiratory viruses, while EV-A (*E. alphacoxsackie*) and EV-B (*E. betacoxsackie*) gather enteric viruses. By contrast, the species EV-C (*E. coxsackiepol*) and EV-D (*E. deconjuncti*) contain both respiratory and enteric viruses (6).

In many aspects, the species *E. deconjuncti* is of particular interest (8). First, only five serotypes have been described among this species, whereas the other EV species that infect humans contain dozens of serotypes each. Moreover, the few known EV-Ds differ significantly from one another in terms of tissue tropism: EV-D68 is a respiratory virus, EV-D70 has a strong ocular tropism, while EV-D94, D111, and D120 seem to replicate mainly in the intestine (9–12). It is important to note that this phenotypic diversity do not reflect the phylogenetic relationships between the different EV-Ds. The first identified EV-D was EV-D68, a respiratory virus that was also called Human rhinovirus 87. Although discovered as early as 1962 (13), this virus was not studied for decades since it had a modest impact on human health. In 2014, EV-D68 has occasionally been responsible for severe respiratory distress and has been associated with acute flaccid myelitis cases (14, 15). The reasons why this virus, known since the 1960s, has suddenly become more pathogenic for humans are not yet completely understood (16). The second known EV-D was EV-D70, which emerged in large outbreaks of acute hemorrhagic conjunctivitis in Africa in the late 1960s and then spread worldwide during the 1970s and 1980s (17–20). Serological studies demonstrated the presence of antibodies directed against EV-D70 in sera from domestic animals collected before the epidemics occurred (21, 22). This led to the hypothesis of a zoonotic origin of this virus. This possibility is also supported by the fact that the most recent common ancestor of all known EV-D70s circulated around 1967, just before the first human outbreak in Africa (23). Africa seems to be the cradle of the three other known EV-Ds: although serological studies have detected antibodies against EV-D94 and EV-D111 in humans in different European countries (10, 24, 25), these two viruses have never been detected outside Africa, either by virus isolation or by molecular detection. EV-D94 and EV-D111 have been detected in human stools and in wastewater during poliovirus surveillance programs (10, 11, 26, 27). Interestingly, EV-D111 was also detected in the stools of non-human primates (NHPs) living in remote areas of Central Africa (28, 29). This observation and the fact that EV-D120 was detected in NHP samples, but not in humans, also point to a zoonotic origin of EV-D111 and, possibly, of EV-D94 (26).

**Fig 1:**
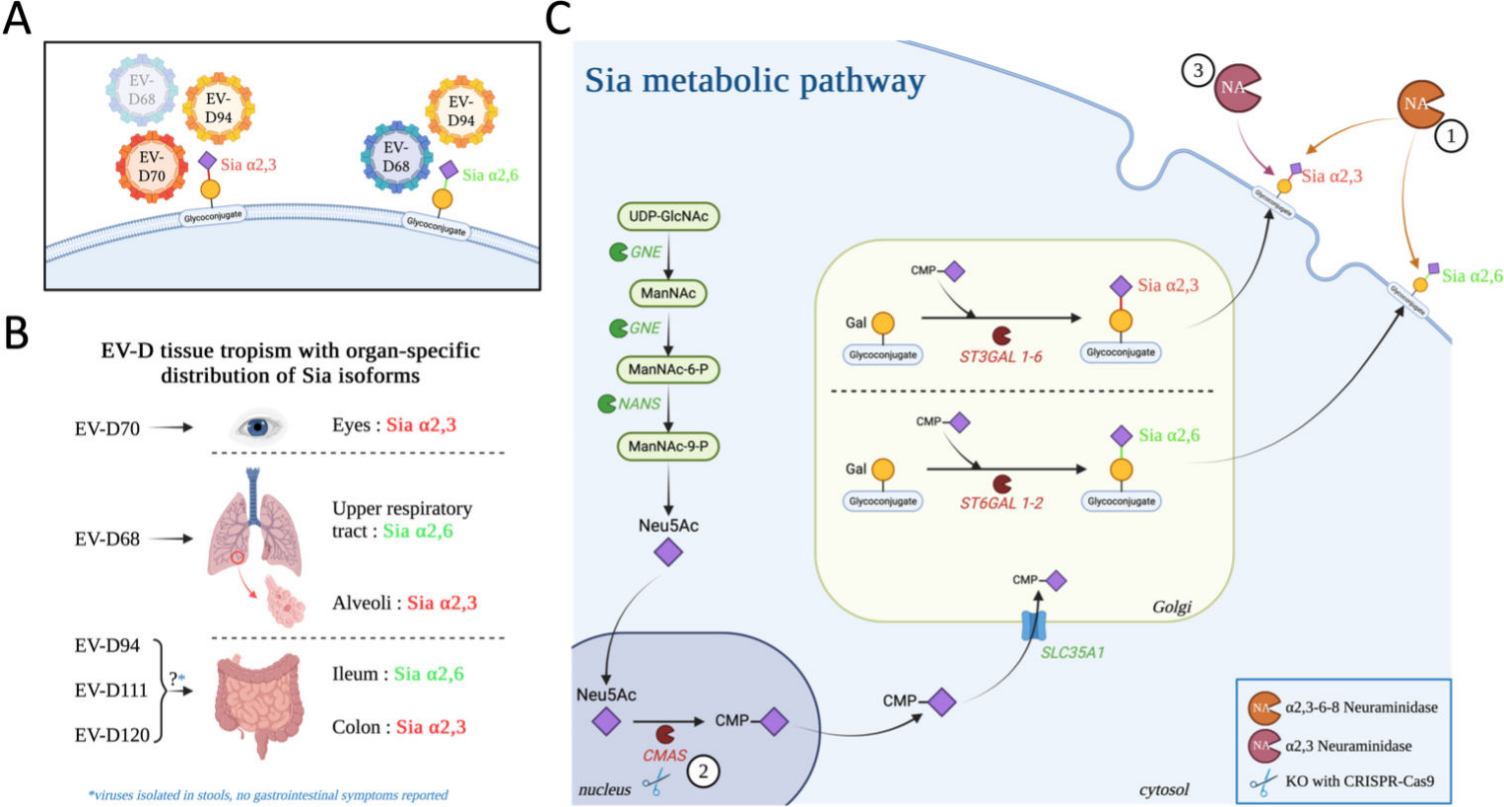
Metabolic pathway of Sia and EV-D usage. (**A**) EV-D70 binds only to Sia α2,3, while EV-D94 uses both isoforms. EV-D68 has a strong preference for Sia α2,6, but is also able to bind to Sia α2,3 with less affinity. (**B**) These isoform preferences correlate with the tropism of these different viruses. (**C**) These Sias are produced by a metabolic pathway involving various key enzymes. GNE catalyses the first step in Sia biosynthesis. Sia is then activated thanks to the action of CMAS (cytidine monophosphate N-acetylneuraminic acid synthetase) by coupling it with a cytidine monophosphate (CMP), producing a CMP-sialic acid. This substrate is then transported to the Golgi apparatus and used by two types of sialyltransferases, ST3GAL1-6 and ST6GAL1-2. Each family proceeds to the sialylation of glycoproteins and produces, respectively, the two major isoforms, Sia α2,3 and Sia α2,6. The glycoconjugates are then exported to the cell surface. Sias can be removed from the surface of the cell thanks to exogenous neuraminidase targeting either a specific isoform (α2,3 NA; third tool; circled number 3) or all sialylated glycoconjugates (α2,3-6-8 NA; first tool; circled number 1). It is also possible to inhibit the production of these glycoconjugates by targeting key enzymes in the Sia biosynthesis pathway, in particular CMAS (second tool; circled number 2). The figure was created with BioRender.

The replication cycle of EV-D94 and EV-D111 is poorly described, and in particular, their entry mechanisms. Cell entry receptors used by various EVs have been identified, mostly belonging to the immunoglobulin or integrin receptor family (30) and induce a conformational change in the capsid, which enables decapsidation and release of the viral genome into the cytoplasm. There are no known receptor(s) for EV-D94 and EV-D111 yet. Attachment factors concentrate viral particles on the cell surface and increase the probability of viruses encountering their entry receptor (31). Previous studies have found that EV-D68 and EV-D70 use sialic acids (Sias) as attachment factors (32–37) (Fig. 1A), which is in line with their respective tropism (12, 38). EV-D68, which is responsible for severe respiratory diseases, preferentially binds Sias α2,6 (34, 35) that are predominant in the upper respiratory tract (39). EV-D70, which causes acute hemorrhagic conjunctivitis (40), uses Sias α2,3 (36), which are abundant in the ocular conjunctival cells (Fig. 1B). The adaptation of EV-D70 to replication in the eyes is not unique in the *Enterovirus* genus. Indeed, coxsackievirus A24 (CVA24, member of the EV-C species) was known for two decades without being associated with any human pathology and was described as an enteric virus. Then, a variant emerged, CVA24v, which is now the major cause of haemorrhagic conjunctivitis and has acquired the ability to bind Sias α2,3, conferring its ocular tropism (41, 42). However, there is conflicting information in the literature regarding the role that certain proteins may play in EV-D68 entry: some reports claim that Sias induce virus decapsidation (35), while others consider them as attachment factors only (30). Besides, it was also shown that EV-D94 uses Sias as attachment factors (35, 37) (Fig. 1A). This phenotypic trait is more surprising since EV-D94 has all the properties of an enteric virus: it was found in stools and the viral particles are not inactivated by acidic pH (10, 43). The use of Sias is rare among EVs, with only two other referenced Sia-using viruses. Besides the example of the specific CVA24v cited above (41), some variants of EV-A71, one of the etiological agents of hand-foot-and-mouth syndrome, use Sias (44). Furthermore, the fact that EV-D68 uses Sias to infect human cells is not surprising, given that these attachment factors are frequently used by respiratory viruses (39, 45–47). On the other hand, the fact that EV-D70, which replicates in the eyes, and EV-D94, which possesses characteristics of enteric viruses, also uses Sias to infect cells raises more questions (Fig. 1B).

Previous studies on EV-D111 did not address its ability to bind Sias. We wanted to determine whether this phenotypic trait, already described for EV-D94, was shared with EV-D111. We used exogenous neuraminidase treatments and generated knockout cells to target different steps of the Sia biosynthesis pathway (Fig. 1C). Our results show that EV-D111 uses Sias during infection of human and simian cells, while a simian EV belonging to another species, EV-B114, does not. These findings suggest that the use of Sias could be an ancestral trait of all EV-Ds that is linked to their ability to infect NHPs.

## MATERIALS AND METHODS

### Cells and viruses

Human RD muscle cells, derived from rhabdomyosarcoma, were supplied by the *National Institute for Biological Standards and Control* (NIBSC, Potters Bar, UK). They were grown in *Minimum Essential Medium Eagle* (MEM; Gibco) supplemented with 5% (vol/vol) fetal bovine serum (FBS; Gibco) and 2 mM L-Glutamine (Gibco). Simian LLC-MK2 cells from rhesus monkey kidney were from the ATCC. They were grown in *Medium 199* (Gibco) supplemented with 1% (vol/vol) horse serum (Gibco) and 2 mM L-Glutamine (Gibco). These adherent cells were maintained in monolayer culture and divided once at confluence after dissociation by the action of trypsin-EDTA (Gibco). Cell culture was performed at 37°C under 5% CO_2_.

The viruses used in this work belong to the laboratory’s collection. The strain of EV-D111 (CAF-OUP-05-059) used in this study was obtained after three passages on RD cells since isolation in cell culture from stool samples from children with acute flaccid paralysis as part of the poliovirus surveillance (26, 27). The Fermon, J670/71, and Nancy strains were isolated decades ago and are, respectively, the prototypes of EV-D68, EV-D70, and coxsackievirus B3 (CVB3); they are available through the European Virus Archive (48). The strain of the simian enterovirus CMR08-Z057 used in this study belongs to the serotype EV-B114, which was first isolated in the 1950s and previously known as SA5 (49, 50). All works involving infectious materials were carried out in a BSL-2 facility.

### Viral infection

The day before infection, 12-well plates were seeded with RD cells at a concentration of 2.5 × 10^5^ cells/well or 10^5^ cells/well for LLC-MK2 cells. They were then infected with an inoculum without FBS at a multiplicity of infection (MOI) adjusted according to experiments and incubated for 1 h at 37°C under 5% CO_2_. Inoculum was then discarded, and fresh MEM medium supplemented with 2% FBS was added; infection was stopped by freezing. After three freezing/thawing cycles, the cell supernatants were clarified by centrifugation (5 min at 3,000 g) and stored at −80°C until use.

### Sialic acid removal from the surface of cells

Where indicated, cells were treated with neuraminidase (NA) prior to infection to remove Sias from the surface of cells. Two different NAs were used, either targeting specifically Sias α2,3 or all three isoforms indiscriminately (Sias α2,3; α2,6; α2,8). Cells were first washed with phosphate-buffered saline (PBS; Gibco) and then 200 µL of Opti-MEM (Gibco) containing either 31.25 µL of NA α2,3 (250 units; from *Streptococcus pneumoniae*; 8,000 units/mL; New England Biolabs) or 5 µL of NA α2,3-6-8 (250 units; from *Clostridium perfringens*; 50,000 units/mL; New England Biolabs) was spread on the cell monolayer. The cells were then incubated at 37°C under 5% CO_2_ for 1 h before being washed again with PBS, and then infection was carried out as described in the previous paragraph.

### Viral titration

Viral titers were determined by a 10-fold dilution method (10 wells per dilution). A suspension of RD cells at a concentration of 1.2 × 10^5^ cells/mL (150 µL per well) was used to seed a 96-well plate. 50 µL of the different dilutions of the viral sample were added to the wells, and the plate was then incubated for 7 days at 37°C under 5% CO_2_ before determining the cytopathic effect (CPE) by microscopic observation. The median tissue culture infectious dose (TCID_50_) assay was calculated using the Spearman-Kärber method; it corresponds to the inverse of the viral suspension dilution giving a CPE in 50% of inoculated culture wells.

### RNA extraction and real-time RT-PCR analysis

Viral RNA was extracted using the QIAamp Viral RNA Mini Kit (Qiagen) and following the supplier’s instructions. Total nucleic acid was eluted in 60 µL. TaqMan real-time RT-PCR was performed to quantify viral RNA using the Applied Biosystems StepOnePlus Real-Time PCR System (ThermoFisher Scientific) and the SuperScript III Platinum One-Step RT-qPCR Kit (ThermoFisher Scientific). A set of sense and antisense primers (20 µM) and a probe (10 µM) specific to EV-A, EV-B, EV-C, and EV-D were used: sense primer (EV2) 5′-CCCCTGAATGCGGCTAATC-3′, antisense primer (EV1) 5′-GATTGTCACCATAAGCAGC-3′, probe (EV-probe) 5′-[6FAM]CGGAACCGACTACTTTGGGTGTC CGT[TAMRA]-3′. The following program was run for all RT-qPCR reactions: 15 min at 45°C, then 2 min at 95°C, followed by 45 cycles of 15 s at 95°C and 1 min at 60°C. The threshold cycles (Ct) obtained were then converted to the quantity of RNA expressed in Equivalent TCID_50_/mL using a standard curve.

### Generation of knockout RD cells

Cas9-mediated knockout of CMAS was performed using two couples of sgRNAs to target exon 3 (5′-CACCGACCTCCGTCTCCAACCCGCG-3′ and 5′-AAACCGCGGGTTGGAGACGGAGGTC-3′) and exon 8 (5′-CACCGTGCTCTGCAAATTCTCGGA-3′ and 5′-AAACTCCGAGAATTTGCAGAGCAC-3′) of CMAS in combination. The sgRNA sequence was cloned into the vector lentiCRISPRv2 expressing Cas9 protein with a resistance to either puromycin (lentiCRISPR v2, Addgene plasmid #52961, sgRNA targeting exon 3 of CMAS) or blasticidin (lentiCRISPR v2-Blast, Addgene plasmid #83480, sgRNA targeting exon 8 of CMAS). A packaging system with plasmid psPAX2 encoding the structural protein and plasmid pMD2.G encoding the VSV-G envelope protein was utilized to produce lentiviral particles. 6-well plates were seeded with 2 × 10^6^ cells/well of HEK 293T the day prior to infection. The polymer reagent X-tremeGENE9 (Roche) was used to co-transfect 1.6 µg of lentiCRISPRv2 with sgRNA, 1 µg of psPAX2, and 0.4 µg of pMD2.G. At 48 h post-transfection, the supernatant was collected and filtered through a 0.45 µm filter (Whatman). RD cells in a 6-well plate were transduced with lentivirus using polybrene (Sigma-Aldrich) at a final concentration of 8 µg/mL and then incubated at 37°C for 72 h before selection with puromycin (0.5 µg/mL) and blasticidin (3 µg/mL). Control RD cells were generated with the same protocol using non-targeting lentiCRISPR v2-sgControl (Addgene plasmid #125836), followed by puromycin selection (0.5 µg/mL).

### Western blot

Cells were lysed in radioimmunoprecipitation assay (RIPA) buffer (Sigma-Aldrich) supplemented with a protease and phosphatase inhibitor cocktail (Roche). Supernatants were denatured in 4× Protein Sample Loading Buffer (Li-Cor Bioscience) under reducing conditions (NuPAGE Sample Reducing Agent; Invitrogen) at 70°C for 10 minutes. Proteins were separated by SDS-PAGE (NuPAGE 4–12% Bis-Tris Gel; Invitrogen) and transferred to nitrocellulose membranes (Bio-Rad) using a Trans-Blot Turbo Transfer system (Bio-Rad). Membranes were blocked for 1 h with PBS-0.1% Tween 20 (PBS-T) containing 5% milk and were incubated sequentially with rabbit polyclonal anti-CMAS (1:1,000, HPA039905; Sigma-Aldrich) and rabbit polyclonal anti-GAPDH (1:20,000, 10494-1-AP; proteintech) diluted in blocking buffer overnight at 4°C. The membranes were then incubated with secondary antibodies (goat anti-rabbit IgG (H + L) DyLight 800 and then donkey anti-rabbit IgG (H + L) Alexa Fluor 680; ThermoFisher Scientific) diluted in blocking buffer for 1 h at room temperature. Images were acquired using an Odyssey CLx infrared imaging system (Li-Cor Bioscience), and protein levels were quantified using Image Studio Software.

### Immunofluorescence assays

Infected cells were washed twice in PBS at, respectively, 6- or 8 hours post-infection and then fixed with 4% paraformaldehyde (PFA; Sigma-Aldrich) for 10 min at room temperature (RT). Fixed cells were then permeabilized with PBS containing 0.5% Triton (Sigma-Aldrich) for 15 min and blocked for 30 min at RT with PBS containing 0.05% Tween and 5% bovine serum albumin (BSA; Sigma-Aldrich). Cells were stained for 1 h with a rabbit anti-VP1 polyclonal antibody specific for EV-D68 (1:1,000, GTX132313; GeneTex) and a mouse anti-dsRNA monoclonal antibody (1:200, MABE1134; Sigma-Aldrich). Cells were washed three times with PBS before incubation with secondary antibodies (Alexa Fluor 488 goat anti-rabbit and Alexa Fluor 647 donkey anti-mouse, A11008, A31571; ThermoFisher Scientific) for 45 min at RT. Nuclei were stained with Hoechst (1:10,000; ThermoFisher Scientific). After three washes with PBS, slides were mounted with ProLong Gold antifade reagent (P36930; Life Technologies). Images were acquired using a confocal microscope (Leica TCS-SP8). The number of nuclei and infected cells was quantified using Fiji.

### Virus binding assays

The day before performing binding assays, 12-well plates were seeded with 2.5 × 10^5^ RD cells/well. Where indicated, cells were treated with neuraminidase (NA) prior to infection. EV-D111 (MOI of 10) was added to the pre-chilled cells and incubated on ice for 15 min. To remove unbound virions, cells were washed six times with ice-cold PBS supplemented with 2% BSA. Cells were lysed directly in the plate by adding 350 µl RLT Buffer with β-mercaptoethanol and collected for RNA extraction using the RNeasy Mini Kit (Qiagen) and following the supplier’s instructions. TaqMan real-time RT-PCR was performed to quantify viral RNA using the Applied Biosystems StepOnePlus Real-Time PCR System (ThermoFisher Scientific) and the SuperScript III Platinum One-Step RT-qPCR Kit (ThermoFisher Scientific). Primers and probes used are as follows: EV-D111_Fw: 5′-CCAGAACAAGCAGCCAACAC-3′, EV-D111_Rv: 5′-CTGTATAGCTTCCTCTGTGC-3′, EV-D111_probe: 5′-[6FAM]GTGGCTAACACAATAGAGAGTGAGG[BHQ1]-3′, GAPDH_Fw: 5′-GTCTCCTCTGACTTCAACAGCG-3′, GAPDH_Rv: 5′-ACCACCCTGTTGCTGTAGCCAA-3′, GAPDH_probe: 5′-[6FAM]TGACGCTGGGGCTGGCAT TGC[BHQ1]−3′. Data were analyzed using the 2^−ΔΔCt^ method, with all samples normalized to endogenous GAPDH.

### Transfection of viral RNA

To ensure that the WT and KO cell lines were transfected with the same efficiency, we first performed an assay with an *in vitro* transcript obtained from the plasmid pFULL-Luc (available through the European Virus Archive portal). It contains the nanoluciferase gene (with start and stop codons) flanked by genomic regions of a coxsackievirus B4 (strain C08-219 (27)): the 5′UTR, the 2C-encoding sequence, and the CVB4 3′UTR, including the poly-A tail. As the EV 5′UTR contains an Internal Ribosome Entry Site (IRES) that enables cap-independent translation, pFULL-Luc contains all the elements needed for the translation of the nanoluciferase gene in mammalian cells. The day before transfection, 96-well plates were seeded with 5 × 10^4^ cells/well, and *in vitro* transcripts were transfected using Lipofectamine (ThermoFisher Scientific) following the manufacturer’s instructions. The cells were then cultured for 16 h before revealing luminescence with the Nano-Glo Luciferase Assay System (Promega) following the supplier’s instructions. Luminescence was assessed with the TECAN Infinite M200 Pro (5,000 ms acquisition).

WT and KO cells were transfected with 5 µL of EV-D111 viral RNA (15.5 ng/µL) using Lipofectamine (ThermoFisher Scientific). vRNA was extracted from EV-D111-infected RD cells. The day before transfection, 48-well plates were seeded with 1.5 × 10^5^ RD cells/well in MEM medium supplemented with 2% FBS. The cells were then incubated at 37°C under 5% CO_2_, and viral production was stopped by freezing at different time post-transfection. After three freezing/thawing cycles, the cell supernatants were clarified by centrifugation (5 min at 3,000 g) and stored at −80°C until viral titration.

## RESULTS

### EV-D111 uses Sias to infect human RD cells

To investigate the use of Sias during EV-D111 infection, different steps of the Sia biosynthesis pathway were targeted (Fig. 1C). Sias can be removed from the surface of cells using exogenous neuraminidase targeting either all sialylated glycoconjugates (α2,3-6-8 NA; Fig. 1C, first tool) or a specific isoform (α2,3 NA; Fig. 1C, third tool). It is also possible to inhibit the production of these glycoconjugates targeting key enzymes of the pathway using the CRISPR-Cas9 method, in particular cytidine monophosphate N-acetylneuraminic acid synthetase (CMAS) (Fig. 1C, second tool).

The importance of Sias during infection was first investigated by treatment with NA, a sialydase that removes Sias α2,3, α2,6, and α2,8 from the cell surface (Fig. 1C, first tool). Since EV-D70 uses Sias to infect HeLa (cervical carcinoma), U-937 (human histiocytic lymphoma), and NIH 3T3 (murine fibroblast) cells (36), this virus was used as a positive control. CVB3, which enters cells independently of Sias (35), was used as a negative control. RD cells previously treated with NA were infected with EV-D111, EV-D70, or CVB3 at high MOI (MOI 10) to ensure initial infection of all cells. Viral replication was assessed at different times post-infection (0, 2, 4, 8, 11, and 24 hours post-infection [hpi]) by quantifying production of viral RNA by RT-qPCR and infectious viral particles by viral titration. The threshold cycles (Ct) obtained by RT-qPCR were then converted to the quantity of RNA expressed in Equivalent TCID_50_/mL using a standard curve.

**Fig 2:**
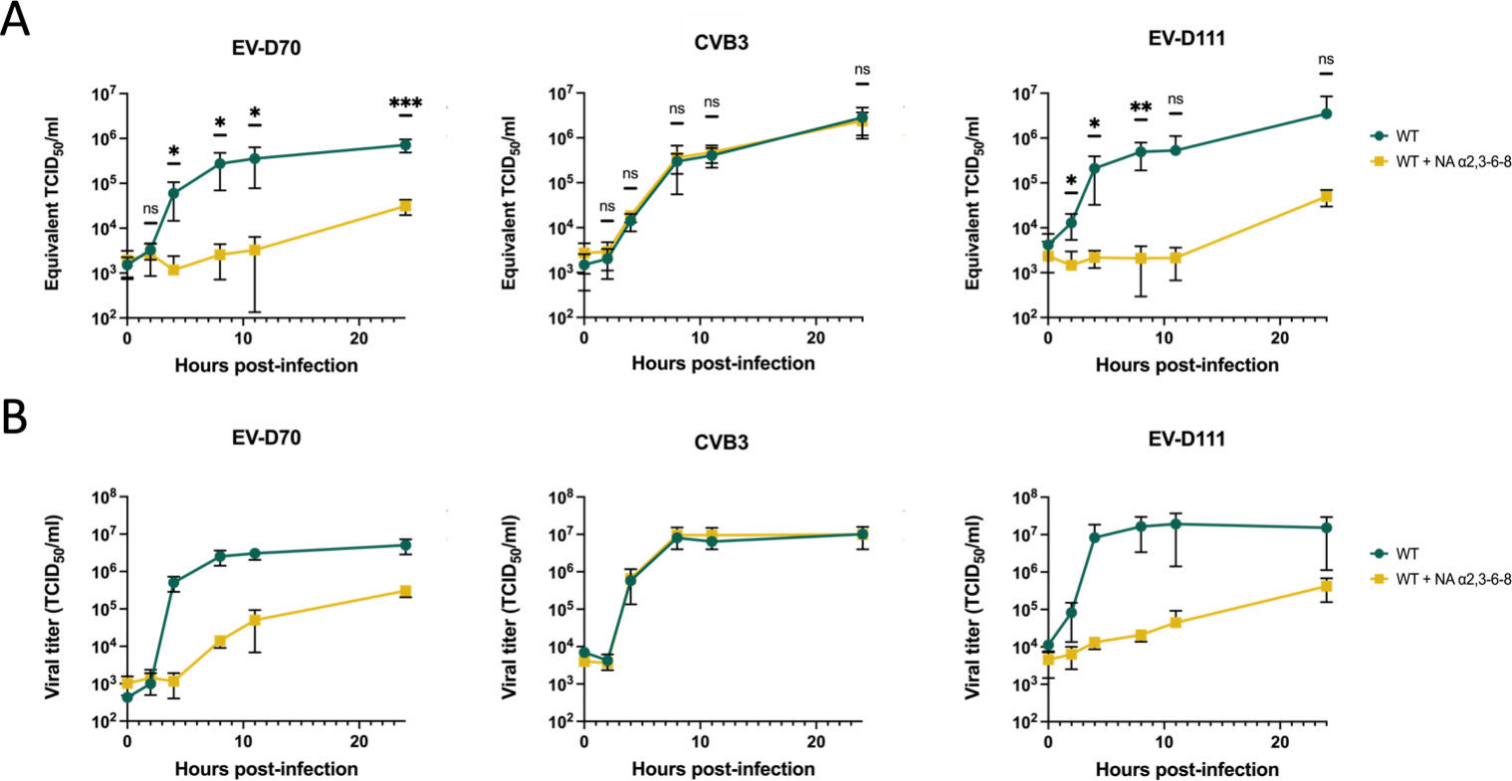
Enzymatic treatment shows that EV-D111 uses Sia to infect human RD cells. RD cells treated (WT +NA α2,3–8) or not (WT) with NA removing α2,3- α2,6-, and α2,8-linked Sia were infected with EV-D70, CVB3, or EV-D111 at an MOI of 10, ensuring a single cycle of virus production. After three freeze-thaw cycles, well contents were harvested and clarified. (**A**) Viral RNA production during infection (at 0, 2, 4, 8, 11, and 24 hpi) was detected through real-time RT-PCR. Cycle threshold (Ct) values were converted to Equivalent TCID_50_/mL using a standard curve. The individual points represent the mean ± IC95% of three biological replicates and three technical replicates each. *P* values were calculated by two-way ANOVA of log-transformed data with Šidák’s multiple comparison test, *P < 0.05; **P < 0.01; ***P < 0.001; ****P < 0.0001; ns not significant. (**B**) The production of infectious viral particles was quantified by the median tissue culture infectious dose (TCID_50_) assay, determining the concentration at which 50% of infected cells exhibit cytopathic effect (CPE). The mean ± IC95% of three biological replicates is shown.

In untreated cells, production of viral RNA (Fig. 2A) and infectious viral particles (Fig. 2B) increases as early as 4 hpi for all three viruses. A plateau was reached at around 8 hpi. For all three viruses, the titers were close to 10^7^ TCID_50_/mL at the end of the kinetics, and the amount of viral RNA exceeded 10^6^ Equivalent TCID_50_/mL.

As expected (36), the production of EV-D70 RNA and infectious particles was significantly reduced in treated cells, while CVB3 production was unaffected (Fig. 2A and B). These results show that NA treatment effectively degrades Sias present on the cell surface. In treated cells, EV-D111 showed a profile similar to that of EV-D70, with a significant decrease in viral RNA yield and infectious particle production compared to untreated cells (Fig. 2A and B). These results suggested that EV-D111, like EV-D70, utilizes Sias when infecting RD cells.

**Fig 3:**
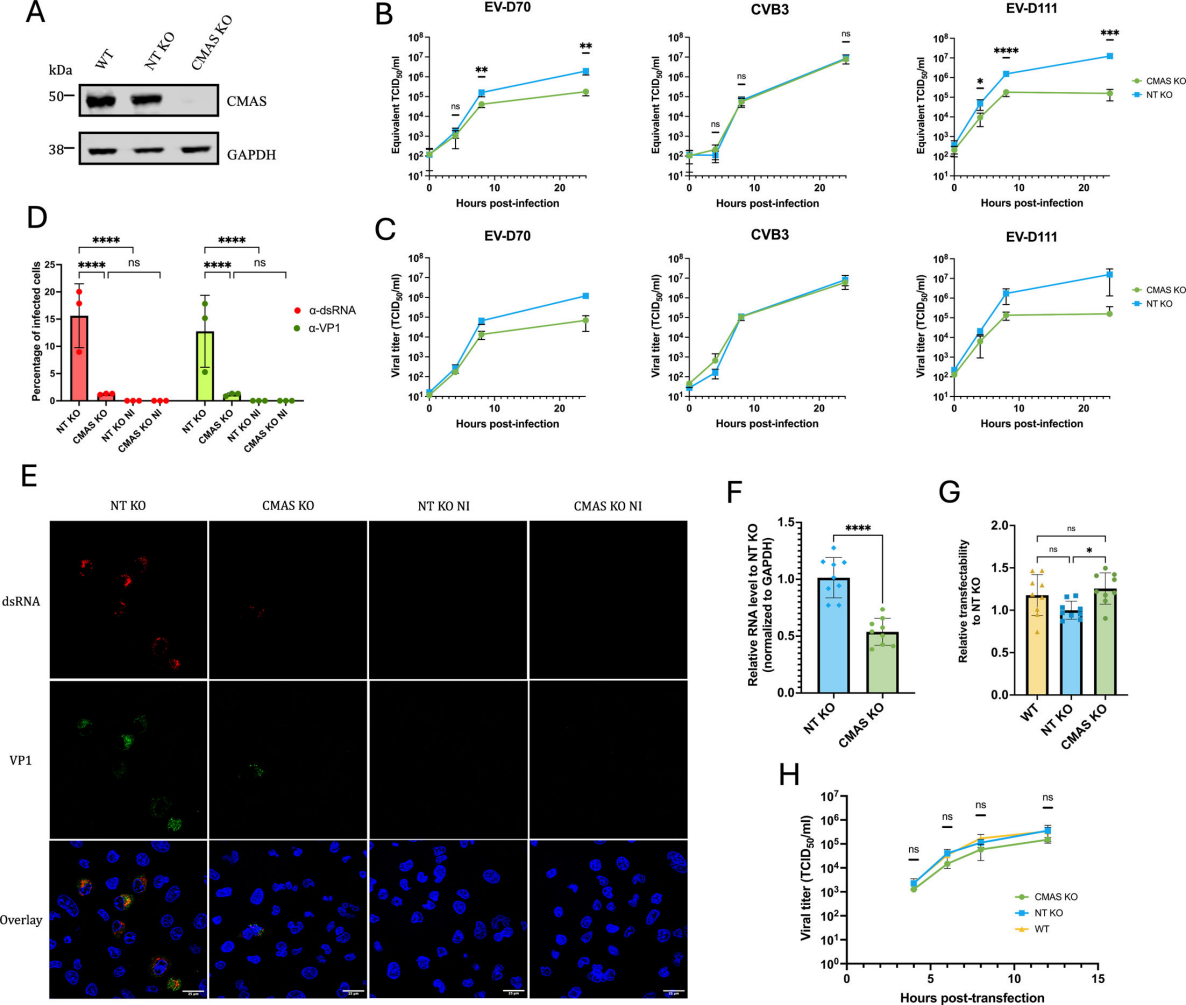
Absence of Sia biosynthesis inhibits EV-D111 infection of RD cells. RD CMAS KO cells (CMAS KO) were produced by transduction with lentiguides targeting the CMAS gene. RD cells transduced with non-targeting lentiguide (NT KO) served as controls. These cells were infected with EV-D70, CVB3, or EV-D111 at an MOI of 0.1. After three freeze-thaw cycles, well contents were harvested and clarified. (**A**) Whole-cell lysates of RD cells (WT), NT KO cells, and RD CMAS KO cells were analyzed by Western blotting with antibodies against the indicated proteins. GAPDH was used as the control marker of protein expression. Data are representative of three biological replicates. (**B**) Viral RNA production during infection (at 0, 4, 8, and 24 hpi) was detected through real-time RT-PCR. Cycle threshold (Ct) values were converted to Equivalent TCID_50_/mL. The individual points represent the mean ± IC95% of three biological replicates and three technical replicates each. *P* values were calculated by two-way ANOVA of log-transformed data with Šidák’s multiple comparisons test, *P < 0.05; **P < 0.01; ***P < 0.001; ****P < 0.0001; ns not significant. (**C**) The production of infectious viral particles was quantified by the median tissue culture infectious dose (TCID_50_) assay, determining the concentration at which 50% of infected cells exhibit cytopathic effect (CPE). The mean ± IC95% of three biological replicates is shown. (**D**) NT KO and CMAS KO cells were infected with EV-D111 at an MOI of 0.1 and stained at 8 hpi with antibodies recognizing dsRNA and VP1. For each biological replicate, five fields were imaged with a confocal microscope. The percentage of infected cells was determined after numbering nuclei and cells that were positive for the two viral markers for each field. Data are presented as mean ± IC95% for three biological replicates. *P* values were calculated by two-way ANOVA with Šidák’s multiple comparisons test, *P < 0.05; **P < 0.01; ***P < 0.001; ****P < 0.0001; ns not significant. (**E**) The presented fields are representative of three biological replicates. The scale bar in each picture represents 25 µm. (**F**) NT KO and CMAS KO cells were subjected to binding assays with EV-D111 at an MOI of 10. Relative viral RNA levels to NT KO cells were measured by RT-qPCR analysis and normalized to GAPDH. Data are representative of three biological replicates with three samples analyzed by replicate. Each graph bar represents the mean ± IC95%. *P* values were calculated by unpaired t test, *P < 0.05; **P < 0.01; ***P < 0.001; ****P < 0.0001; ns not significant. (**G**) The transfectability of the three cell lines was assessed by transfecting an *in vitro* transcript that contains all the elements needed for the translation of the nanoluciferase gene in mammalian cells. NT KO cells served as control cells to determine the relative transfectability of each cell line. Data are representative of three biological replicates with three wells analyzed by replicate. Each graph bar represents the mean ± IC95%. *P* values were calculated by ordinary one-way ANOVA, *P < 0.05; **P < 0.01; ***P < 0.001; ****P < 0.0001; ns not significant. (**H**) WT, NT KO, and CMAS KO cells were transfected with EV-D111 RNA. The production of infectious viral particles collected at different hpt (4, 6, 8, and 12) was quantified by TCID_50_ assay. The mean ± IC95% of three biological replicates is shown. *P* values were calculated by Tukey’s multiple comparisons test, *P < 0.05; **P < 0.01; ***P < 0.001; ****P < 0.0001; ns not significant.

To validate the data obtained with the enzymatic treatments, which have a temporary effect, cells knocked out for CMAS were generated using the CRISPR-Cas9 method (Fig. 1C, second tool). This enzyme is an interesting target for the study of Sias, since it intervenes before the action of sialyltransferases, which bind Sias α2,3, α2,6, or α2,8 to oligosaccharides prior to their transport to the cell surface (51). Western blot analyses revealed that the abundance of CMAS was reduced by about 91% in KO cells compared to WT cells or cells transduced with non-targeting gRNA (NT KO) (Fig. 3A). RD CMAS KO cells were infected with EV-D70, CVB3, or EV-D111 at an MOI of 0.1. Viral replication was then assessed at different times post-infection (0, 4, 8, and 24 hpi) by measuring viral RNA yield and infectious particle production.

As expected from our previous results (Fig. 2), CMAS KO cells were less susceptible to infection with EV-D70 and EV-D111 than control cells (Fig. 3). Although KO cells got infected and produced infectious viral particles, the amount of viral RNA or infectious particles produced was about 2-log higher in control cells compared to CMAS KO cells for EV-D111 (Fig. 3B and C). By contrast, CVB3 replication was not affected by reduced levels of CMAS. These results confirm that EV-D111, like EV-D70, uses Sias to infect human RD cells.

Microscopy analyses were used to evaluate the percentage of NT KO and CMAS KO cells infected with EV-D111 (MOI of 0.1) at 8 hpi (Fig. 3D and E). Cells were stained with antibodies against dsRNA, which are produced during EV replication, and against the capsid VP1. Cells positive for the two signals were considered infected (Fig. 3D and E). Approximately 15% of NT KO cells were infected at 8 hpi, while only 1% of the CMAS KO was infected (Fig. 3D), confirming that CMAS KO cells were refractory to EV-D111 infection.

To determine whether virus binding is reduced in the absence of CMAS, binding assays were performed with EV-D111 at an MOI of 10. Experiments were performed at 4°C to allow binding of the virus particles to the cells but not entry. Cell monolayers were extensively washed to remove unbound virus particles. Quantification of viral genome copy number by RT-qPCR analysis was used as a proxy for the amount of virus particles that were bound to the cells. Twice fewer virus particles remained attached to CMAS KO cells as to NT KO cells (Fig. 3F), indicating that the absence of Sia at the cell surface significantly reduced EV-D111 binding to the cells.

To assess whether other steps of the viral cycle were affected by a reduced expression of CMAS, EV-D111 RNA was transfected into CMAS KO cells or control cells, and the production of infectious viral particles was estimated by viral titration. The relative transfectability of the different cell lines was first assessed using an *in vitro* transcript coding for the nanoluciferase gene (Fig. 3G). Despite a statistical difference in the level of luminescence, the signal was in the same order of magnitude in WT, NT KO, and CMAS KO cells, indicating that the three cell lines had similar levels of transfectability. Upon transfection of CMAS KO cells with EV-D111 RNA, infectious particles were recovered as early as 4 hpt and increased over time, revealing that these cells sustained EV-D111 replication (Fig. 3H). Moreover, there was no significant difference in viral production between the different cell lines at the selected time points, demonstrating that the reduced EV-D111 production in CMAS KO cells (Fig. 3B and C) is due to a defect in viral entry.

### EV-D111 seems to bind specifically to the Sias α2,3 to infect RD cells

There is a strong correlation between Sias isoform specificity and EV-D tissue tropism (12, 38). EV-D70 causes acute haemorrhagic conjunctivitis (40) and is known to use Sias α2,3 (36), an isoform predominant in the eyes, while EV-D68, which has a respiratory tropism, preferentially binds Sias α2,6 (34, 35) that are predominant in the upper respiratory tract (39) (Fig. 1A and B).

**Fig 4:**
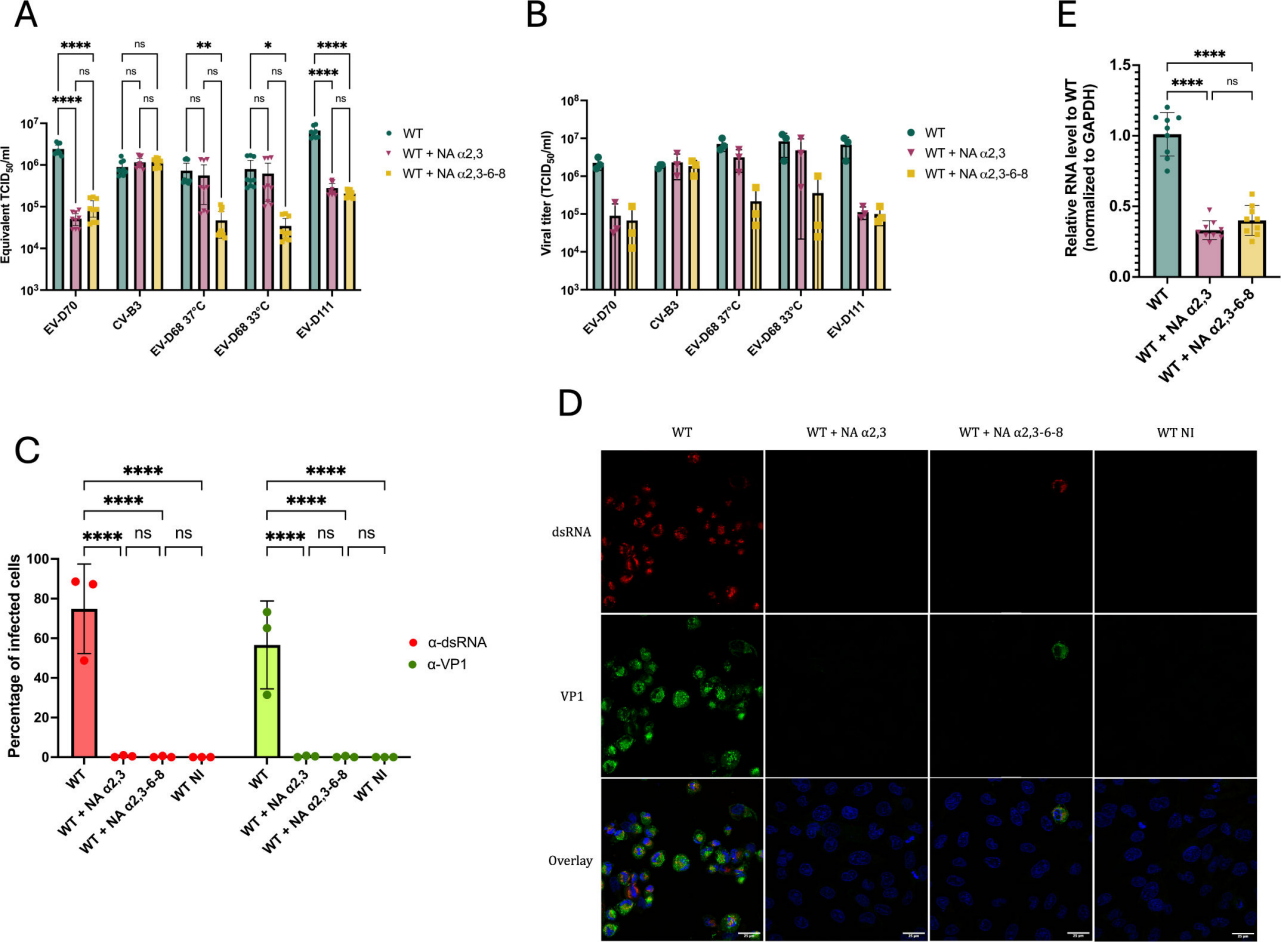
Enzymatic removal of Sia α2,3 reduces infection of human RD cells by EV-D111. RD cells were treated with two different NAs, one targeting specifically Sia α2,3 (NA α2,3) and the other removing all the isoforms (NA α2,3-6-8). Treated and non-treated cells were infected with EV-D70, CVB3, and EV-D68 at two different temperatures or EV-D111 at an MOI of 10. After three freeze-thaw cycles, well contents were harvested and clarified. (**A**) Viral RNA after a single cycle of virus production (8 hpi) was detected through real-time RT-PCR. Cycle threshold (Ct) values were converted to Equivalent TCID_50_/mL using a standard curve. The error bars indicate the 95% confidence intervals calculated on three biological replicates and three technical replicates each. *P* values were calculated by two-way ANOVA of log-transformed data with Tukey’s multiple comparison test, *P < 0.05; **P < 0.01; ***P < 0.001; ****P < 0.0001; ns not significant. (**B**) The production of infectious viral particles was quantified by the median tissue culture infectious dose (TCID_50_) assay, determining the concentration at which 50% of infected cells exhibit cytopathic effect (CPE). The error bars indicate the 95% confidence intervals calculated on three biological replicates. (**C**) Cells, treated with each one of the NA, or not, were infected with EV-D111 at an MOI of 10 and stained at 6 hpi with antibodies recognizing dsRNA and VP1. For each biological replicate, five fields were imaged with a confocal microscope. The percentage of infected cells was determined after numbering nuclei and cells positive for the two viral markers for each field. Data are presented as mean ± IC95% for three biological replicates. *P* values were calculated by two-way ANOVA with Šidák’s multiple comparisons test, *P < 0.05; **P < 0.01; ***P < 0.001; ****P < 0.0001; ns not significant. (**D**) The presented fields are representative of three biological replicates. The scale bar in each picture represents 25 µm. (**E**) WT cells and RD cells treated with the two different NAs were subjected to binding assays with EV-D111 at an MOI of 10. Relative viral RNA levels to WT cells were measured by RT-qPCR and normalized to GAPDH. Data are representative of three biological replicates with three wells analyzed by replicate. Each graph bar represents the mean ± IC95%. *P* values were calculated by ordinary one-way ANOVA with Tukey’s multiple comparisons test, *P < 0.05; **P < 0.01; ***P < 0.001; ****P < 0.0001; ns not significant.

The preference of EV-D111 for one of the isoforms was investigated using an NA that specifically targets Sias α2,3 (NA α2,3) (Fig. 1C, third tool). The NA that removed all the isoforms from the surface of cells (NA α2,3-6-8) was also included in the analysis (Fig. 1C, first tool). Treated and untreated cells were then infected at an MOI of 10 with our three viruses of interest. EV-D70, which uses only Sias α2,3 (36), and CVB3 served as positive and negative control viruses, respectively (Fig. 1A). EV-D68 was added in the analysis since it binds to Sias α2,6. Infection with EV-D68 was performed at either 37°C or 33°C (Fig. 4A and B) since this virus is known to have a respiratory tropism (13) and is sometimes studied at 33°C (37). Thus, we tested the two different temperatures to investigate whether it could influence the infectious profile of EV-D68 when Sias are removed from the cell surface. The viral production was estimated after a single cycle of infection, at 8 hpi. Both treatments reduced the production of EV-D70 and EV-D68 but with different patterns (Fig. 4A and B). For EV-D70, the reduction was similar for both treatments. This was expected since this virus does not use Sias α2,6 (36). By contrast, NA α2,3-6-8 treatment reduced EV-D68 RNA production more than the NA α2,3 treatment did. This is in accordance with previous data showing that EV-D68 preferentially binds Sias α2,6 but can also use Sias α2,3 (35). EV-D111 featured the same profile as EV-D70, with no difference in viral production between the two NA treatments. This result strongly suggested that EV-D111, like EV-D70, uses Sias α2,3 and not Sias α2,6 to infect RD cells.

Microscopy analyses of cells infected with EV-D111 (MOI of 10) were performed using anti-dsRNA and anti-VP1 antibodies at 6 hpi (Fig. 4C and D). Approximately 80% of WT cells were infected while almost no NA-treated cells were positive for viral markers (Fig. 4C), confirming that, upon removal of Sias from the cell surface, cells were no longer susceptible to EV-D111 infection.

Binding assays were performed with EV-D111 after both treatments to further validate the role of Sias α2,3 in viral binding. Three times fewer viral particles were attached to WT cells after removal of this specific isoform than to untreated cells (Fig. 4E), confirming that the absence of Sias α2,3 at the cell surface significantly reduced cell binding of EV-D111. Moreover, NA α2,3-6-8 treatment had the same impact on EV-D111 binding as the specific NA treatment (Fig. 4E), confirming previous results (Fig. 4A and B) and strengthening the conclusion that EV-D111 bound Sias α2,3 but not Sias α2,6.

### Replication of the simian enterovirus EV-B114 is not dependent on Sias

The detection of EV-D111 and EV-D120, another member of the species (26), in stools of NHP living in remote areas of the tropical forest suggests that EV-Ds that circulate among humans have a zoonotic origin (28, 29). The use of Sias by all known EV-Ds could then be an adaptation to their original host, which could be NHPs. We thus investigated whether the use of Sias may be a common trait shared by simian enteroviruses. We studied the simian enterovirus EV-B114 (formerly known as SA5 (52)), which belongs to the EV-B species (50, 53). Only a few specimens of this virus type have been reported, all from NHPs: *Mandrillus sphinx* (54, 55) and *Cercopithecus aethiops* (50). The strain we used was sampled in Cameroon in 2008 (55).

**Fig 5:**
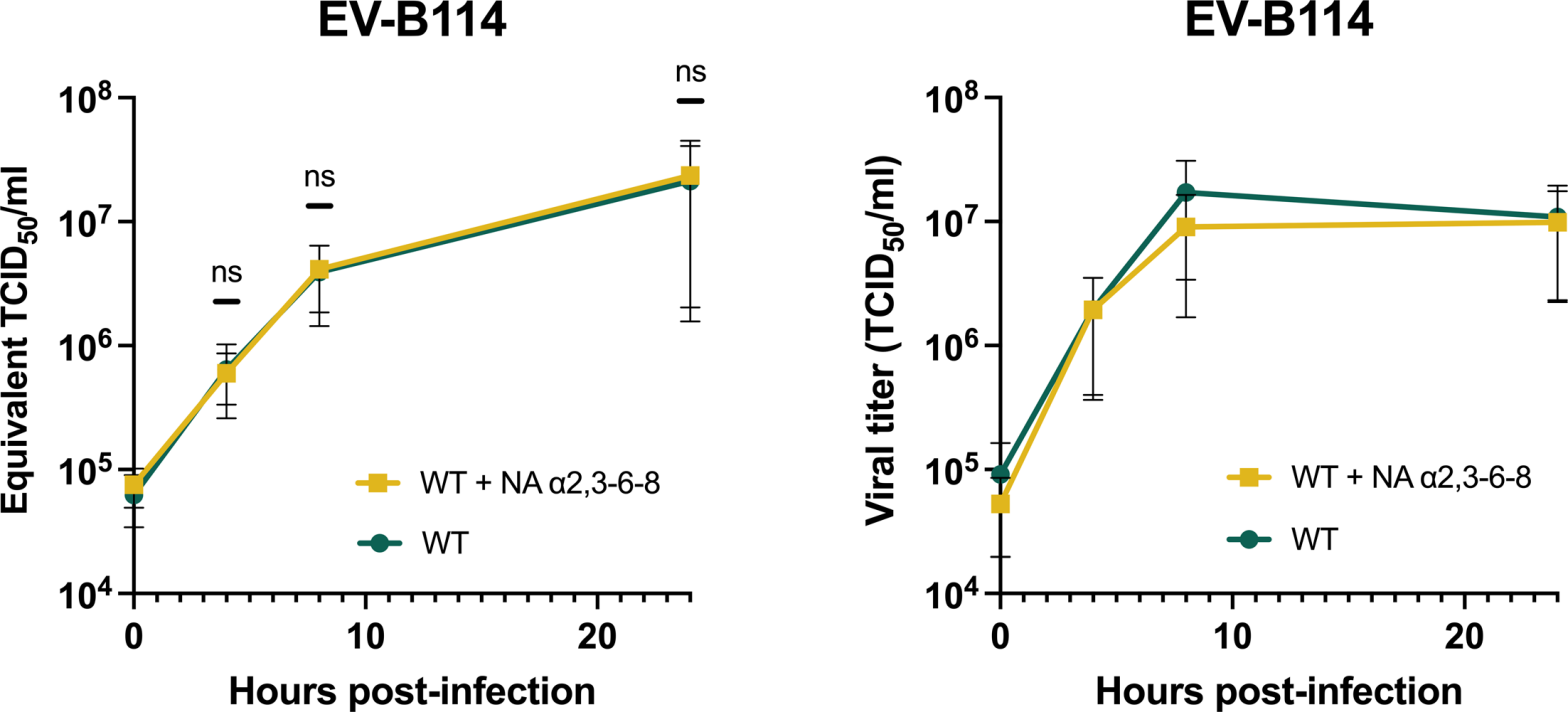
EV-B114 infection is not sensitive to NA treatment in human RD cells. RD cells treated with NA removing α2,3- α2,6-, and α2,8-linked Sia were infected with EV-B114 at an MOI of 10, ensuring a single cycle of virus production. After three freeze-thaw cycles, well contents were harvested and clarified. (**A**) Viral RNA production during infection (at 0, 4, 8, and 24 hpi) was detected through real-time RT-PCR. Cycle threshold (Ct) values were converted to equivalent TCID_50_/mL. The individual points represent the mean ± IC95% of three biological replicates and three technical replicates each. *P* values were calculated by two-way ANOVA of log-transformed data with Šidák’s multiple comparison test; ns not significant. (**B**) The production of infectious viral particles was quantified by the median tissue culture infectious dose (TCID_50_) assay, determining the concentration at which 50% of infected cells exhibit cytopathic effect (CPE). The mean ± IC95% of three biological replicates is shown.

RD cells pre-treated with the NA that removed all the Sias isoforms from the surface of cells were infected with EV-B114 at an MOI of 10 (Fig. 5). Viral RNA yield and production of infectious viral particles were assessed at different time post-infection (0, 4, 8, and 24 hpi). There was no significant difference in viral replication in treated and untreated cells (Fig. 5A). EV-B114 is thus not sensitive to NA treatment, suggesting that the virus does not rely on Sias to enter RD cells.

**Fig 6:**
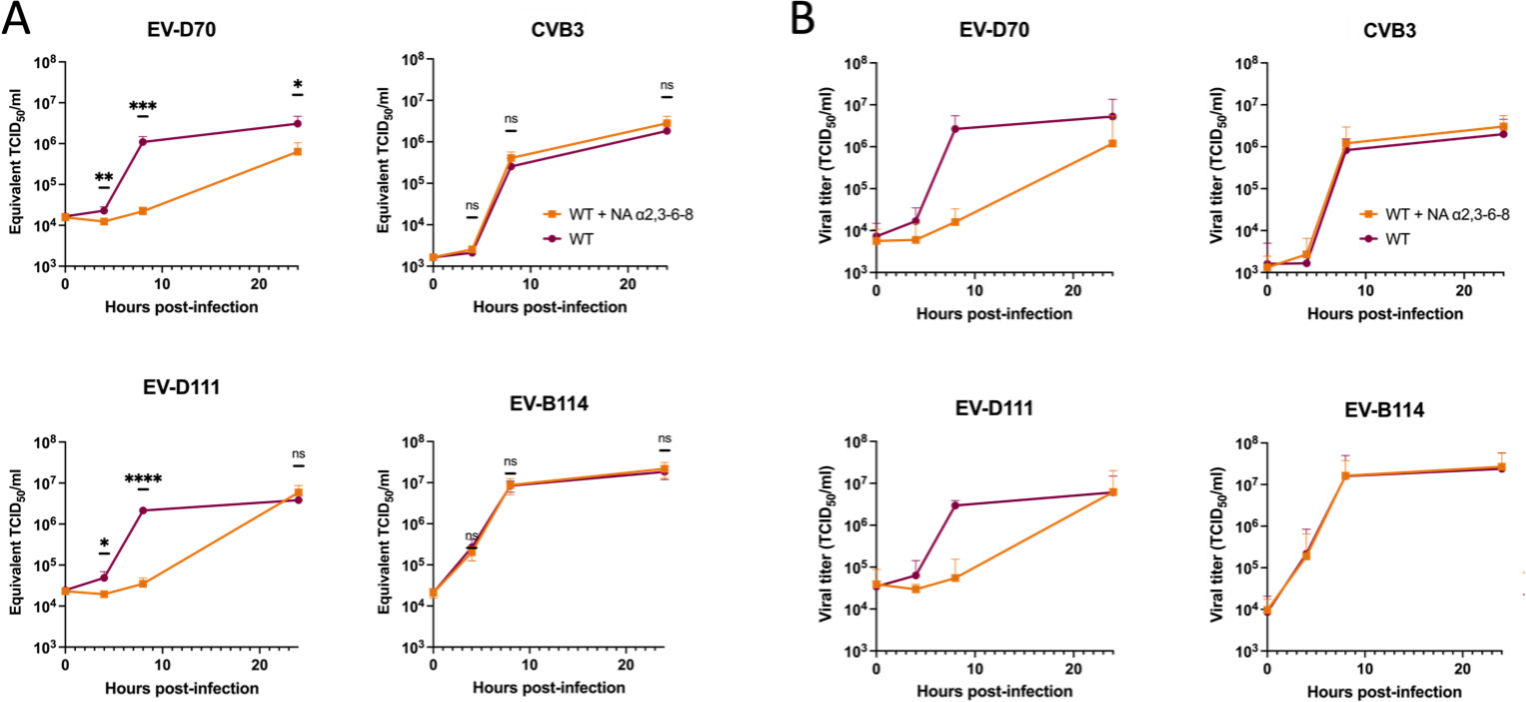
EV-B114 does not use Sia to infect simian LLC-MK2 cells, unlike EV-D70 and EV-D111. LLC-MK2 cells treated with NA removing α2,3- α2,6-, and α2,8-linked Sia were infected with EV-D70, CVB3, EV-D111, or EV-B114 at an MOI of 10, ensuring a single cycle of virus production. After three freeze-thaw cycles, well contents were harvested and clarified. (**A**) Viral RNA production during infection (at 0, 4, 8, and 24 h post-infection) was detected through real-time RT-PCR. Cycle threshold (Ct) values were converted to Equivalent TCID_50_/mL. The individual points represent the mean ± IC95% of three biological replicates and three technical replicates each. *P* values were calculated by two-way ANOVA of log-transformed data with Šidák’s multiple comparison test, *P < 0.05; **P < 0.01; ***P < 0.001; ****P < 0.0001; ns not significant. (**B**) The production of infectious viral particles was quantified by the median tissue culture infectious dose (TCID_50_) assay, determining the concentration at which 50% of infected cells exhibit cytopathic effect (CPE). The mean ± IC95% of three biological replicates is shown.

To confirm these results on simian cells, LLC-MK2 cells, which are derived from the kidney of a rhesus macaque (*Macaca mulatta*), were infected with EV-D70, CVB3, EV-D111, or EV-B114 (Fig. 6). In untreated cells, production of viral RNA (Fig. 6A) and infectious viral particles (Fig. 6B) increased as early as 4 hpi for all viruses, and a plateau was reached at about 8 hpi. For all four viruses, the titers were close to or higher than 10^6^ TCID_50_/mL at 24 hpi, in agreement with the amount of RNA, which exceeded 10^6^ Equivalent TCID_50_/mL. As expected from the previous results (Fig. 2), NA treatment significantly inhibited infection of EV-D70 and EV-D111, with approximately a 2-log difference in viral RNA production between treated and untreated cells at 8 hpi (Fig. 6A). By contrast, the removal of Sias from the surface of cells had no effect on the entry of CVB3 and EV-B114 (Fig. 6). These results show that, as in human cells, EV-D111 and EV-D70 infection depend on Sias in simian cells. They also show that EV-B114 infection in human or simian cells is independent of Sias. Thus, the use of Sias to bind cells is not a common trait shared by all simian or simian ancestor-derived enteroviruses.

**Fig 7:**
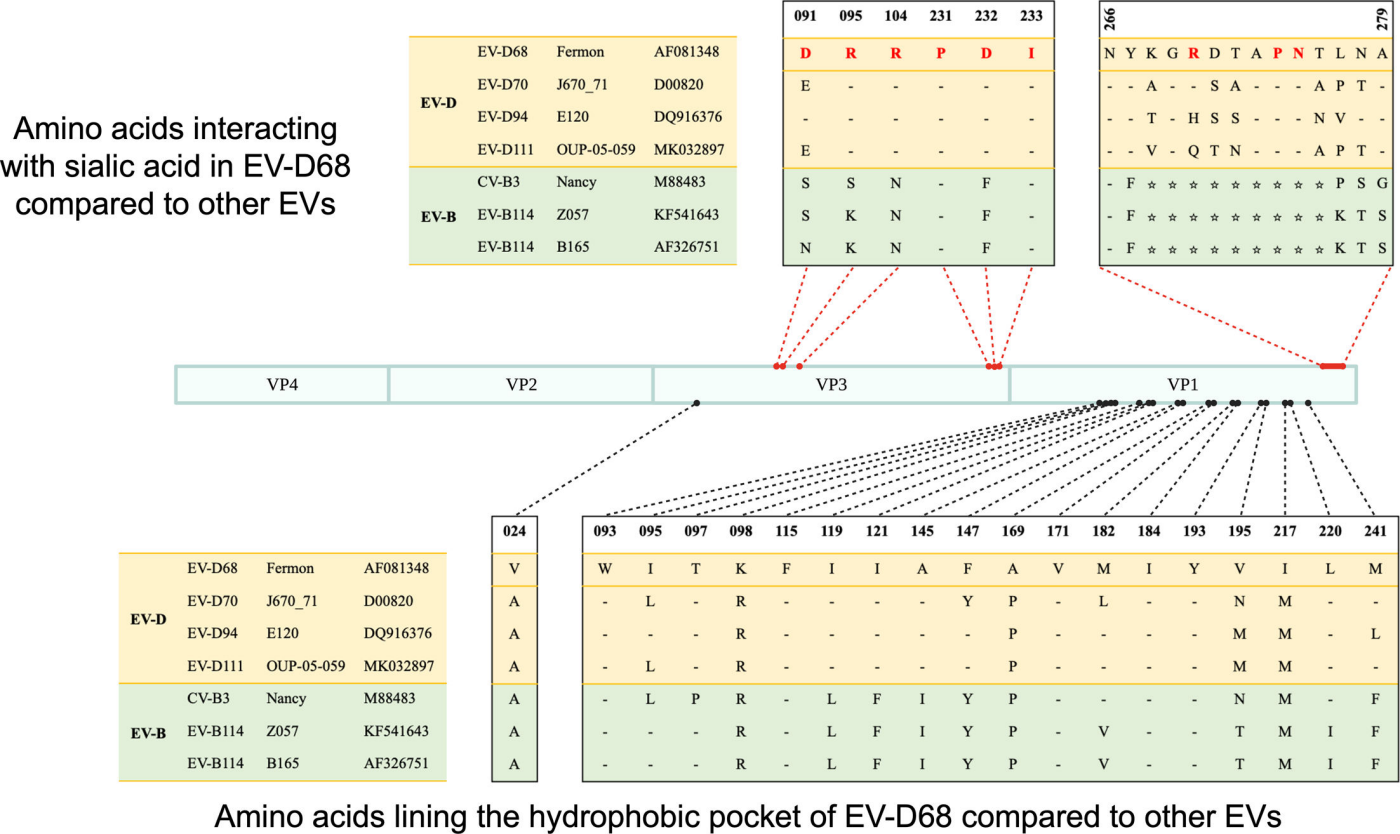
Alignment of amino acids in specific sites of EV-D68 capsid and comparison with other EVs. The residues interacting with the sialic acid moiety (Neu5Ac) and those lining the hydrophobic pocket in EV-D68 were described in reference 34. The capsid sequences of strains used in the present study, belonging either to EV-D or EV-B species, were aligned with CLC Main Workbench 24. GeneBank accession numbers are indicated next to strain names. Above the capsid scheme drawn to scale, amino acids interacting with Sias in EV-D68 (in red) were compared to other EV sequences. Below, residues lining the hydrophobic pocket are listed. « - » represents amino acids identical to the EV-D68 ones, while « ☆ » represents a deletion at a given position. The numbers above the sequences correspond to the amino acid position in the EV-D68 Fermon sequence.

To determine whether the amino acids of the capsid involved in the interaction with the Sias were conserved across EV-Ds and EV-B114, we compared the residues of the four EV-D serotypes and the two EV-B serotypes used in this study with the residues of the EV-D68 Fermon capsid that are known to be involved in stabilizing interactions with the Sia moiety (Neu5Ac) (34). The amino acids lining the hydrophobic pocket appeared to be fairly conserved within the EV-D species (Fig. 7). There were more significant differences between the residues of EV-D68 and those of the EV-B strains. The amino acids involved in the interaction between VP3 from EV-D68 and the Sia moiety are highly conserved in EV-Ds, which is not the case for CVB3 or EV-B114. Only one residue out of six is not conserved, changing from an Asp to a Glu, which is still a diacidic amino acid and retains its physico-chemical properties. Furthermore, there is a deletion of nine residues in the VP1 protein sequence of the two EV-Bs, which coincides with the localization of three residues responsible for Sia attachment in EV-D68. This could explain the absence of interaction of CVB3 and EV-B114 with Sias.

## DISCUSSION

Sias play an important role in the natural history of numerous viruses (47), including naked viruses (*Reoviridae*, *Polyomaviridae*, *Parvoviridae*, and *Adenoviridae*) and enveloped viruses (*Coronaviridae*, *Orthomyxoviridae*, *Paramyxoviridae,* and *Flaviviridae*). The fact that sialylated glycoconjugates served as attachment factors for many unrelated viruses highlights an evolutionary convergence that would confer a considerable gain in fitness (56). This evolutionary advantage is particularly evident for zoonotic viruses. Indeed, the two major Sias isoforms can be found on the cell surface of many wild and domestic mammals, as well as several bird species. Thus, the high prevalence of Sias in animals, including humans, provides an opportunity for a virus using sialylated glycans to cross the species barrier. While the use of Sias is a rare feature in the *Enterovirus* genus, it has previously been shown that three virus types out of five used Sias within the EV-D species. This raised the question of whether this trait could be shared by all EV-Ds. The universal use of Sias by EV-Ds could be linked to their zoonotic origin, which has already been suggested based on genetic, serological, and epidemiological observations (8, 22, 28, 29).

NA α2,3-6-8 treatment experiments demonstrate the involvement of Sias in EV-D111 infection. Viral production resumed after 24 h in treated cells, likely due to the renewal of Sias on the cell surface. Indeed, enzymatic treatment cleaves Sias exposed on the cell membrane but does not inhibit the production pathway. The rate of biosynthesis of new sialylated glycoconjugates is unknown, and NA thus has a temporary effect. Knockout of CMAS expression by the CRISPR-Cas9 approach guaranteed a more stable reduction of the expression of this enzyme, which is essential to Sia biosynthesis. We chose to work with a bulk of KO cells and not a clonal population to avoid selecting a clone with an atypical infectious behavior. A fraction of CMAS KO cells may thus still be susceptible to infection, which would explain why there is a small percentage of infected cells at 8 hpi. Despite this limitation of the cellular model, our results showed that CMAS KO cells are resistant to EV-D111 infection. This confirmed that EV-D111 uses Sias during human cell infection. Thus, EV-D111 exhibits similar Sia usage to EV-D68, EV-D70, and EV-D94 (35, 36). Therefore, all EV-Ds tested so far bind Sias. Unfortunately, EV-D120, the last member of the species, could not be studied as no infectious isolate is available yet. It has only been detected through partial genetic sequences obtained from simian stool samples (28, 29). Rescuing infectious EV-D120 particles from synthetic genomes is unfortunately not an option since the genetic sequences that are available only span the VP4 and the VP1-encoding regions. However, our results suggest that the use of Sias is an ancestral trait of EV-Ds. This seems more likely than an independent acquisition of a derived state for each member of EV-Ds. Given that the EV-D111 strain we used has undergone only three passages in cell culture since its isolation from human stools, we can rule out cell culture adaptation of the strain, as is sometimes the case with some *Picornaviridae*. Indeed, Foot-and-Mouth Disease Virus acquired alternative attachment factors upon adaptation in cell culture, such as heparan sulfate or proteoglycans (57–60). Moreover, EVs are commonly isolated on RD cells, and no selection pressure has been reported for Sia usage for viruses grown this way after a low number of passages. Thus, EV-D111’s ability to bind Sias does not appear to be the result of cell culture adaptation, but rather a feature already present during infection of its natural host. The fact that other EV-D serotypes also depend on Sias for viral replication supports this conclusion.

This is a rather unique trait within the *Enterovirus* genus, since only two other non-respiratory EVs are known to use Sias: CVA24v (41) and EV-A71 (44). Our data revealed that EV-D111 binds Sias α2,3. Moreover, since the additional removal of Sias α2,6 does not reduce EV-D111 production, it can be assumed that this virus uses mainly Sias α2,3 compared to Sias α2,6. It displays the same infectious profile as EV-D70, while EV-D68 binds both with a preference for Sias α2,6. The only experiment conducted on EV-D94 investigating specific isoforms binding suggested that EV-D94 could use both isoforms but did not demonstrate any preference for one isoform or the other (35). It is important to note that the phylogenetic relationships of these five serotypes, based on the sequences of VP1 (the capsid protein that interacts with entry factors), do not reflect the tropism or isoform preference of each of these viruses (8).

Among the five EV-D serotypes, only EV-D68 and EV-D70 have been identified as responsible for human epidemics. Interestingly, these two serotypes exhibit an “atypical” tissue tropism, the first having a respiratory or nervous tropism and the second an ocular tropism. This tissue preference affects pathologies induced, severe respiratory disease, and hemorrhagic conjunctivitis, respectively. We hypothesize that EV-Ds, like all members of the *Enterovirus* genus, initially had an enteric tropism. But based on a shift in Sia usage and the crossing of the species barrier, they may have acquired the ability to enlarge their tissue tropism (respiratory tract or eye) and thus became pathogenic in humans. A similar evolution path was reported for CVA24 (42). This enteric virus was known for two decades without being associated with human pathology, until the variant CVA24v, which causes hemorrhagic conjunctivitis, emerged. This variant has acquired the ability to bind Sias α2,3, which gives it its ocular tropism (41, 42). Thus, a single substitution in the capsid allowed an enteric virus to replicate in new sites and increased pathogenicity.

The phylogeny of the capsid sequences does not match with EV-D tropism (8). Indeed, although EV-D94 has properties of enteric viruses (its infectious particles are acid-resistant), it is phylogenetically closer to EV-D68 (which is acid-sensitive) than it is to EV-D111. Moreover, EV-D70 (which has a strong ocular tropism) is phylogenetically related to EV-D111 and EV-D120, which were both detected in stools (8, 10, 43). This is in line with the shared ability of EV-D70 and EV-D111 to bind Sias α2,3. After emerging in the late 1960s as an agent of acute hemorrhagic conjunctivitis, EV-D70 was responsible for several outbreaks in the 1970s and 1980s. It has apparently disappeared since it has not been reported since 1999. Conflicting data were reported regarding the circulation of EV-D111: serological studies revealed a high proportion of people with anti-EV-D111 antibodies in different African and European countries (25); yet, this virus has never been sampled outside sub-Saharan Africa despite the EV clinical surveillance implemented in many countries. Even in sub-Saharan Africa, the detection of EV-D111 is rare despite intense poliovirus surveillance, whose algorithm relies on cell lines that amplify EV-D111.

Another interesting feature of EV-Ds is that EV-D111 and EV-D120 have been detected in the stools of wild NHPs (28, 29), suggesting a zoonotic origin with a common simian ancestor. Given that the use of Sias appears to be an ancestral trait, we wondered whether this characteristic was shared by all simian enteroviruses. We showed that EV-B114, a simian virus belonging to the EV-B species (50, 53), does not use Sias to enter human RD or simian LLC-MK2 cells. Thus, the use of Sias is not shared by all simian EVs. We also showed a deletion of nine residues in the VP1 sequence of EV-B114, including three amino acids essential for interaction with Sias. This feature shared by CVB3 could explain why, although EV-B114 is a simian EV, it does not use Sias when infecting human or simian cells. As EV-Bs are mainly human viruses, with the exception of a few serotypes (EV-B110, EV-B112, EV-B113, and EV-B114) (61), it would be interesting to complement these results with viruses belonging to the EV-J species, which is exclusively simian. Unfortunately, we do not possess any infectious EV-J isolates. Phylogenetic analyses of different EV-D111 strains do not support the hypothesis of two lineages of this serotype, one circulating in NHPs and the other in the human population, which suggests recent interspecies transmission (8). The fact that EV-D111 has been found only very rarely in humans would indicate that the establishment of post-spillover human-to-human transmission has not yet occurred. However, contacts between wild primates and humans in a context of growing urbanization increase the risk of EV-D111 emergence in humans.

**Fig 8:**
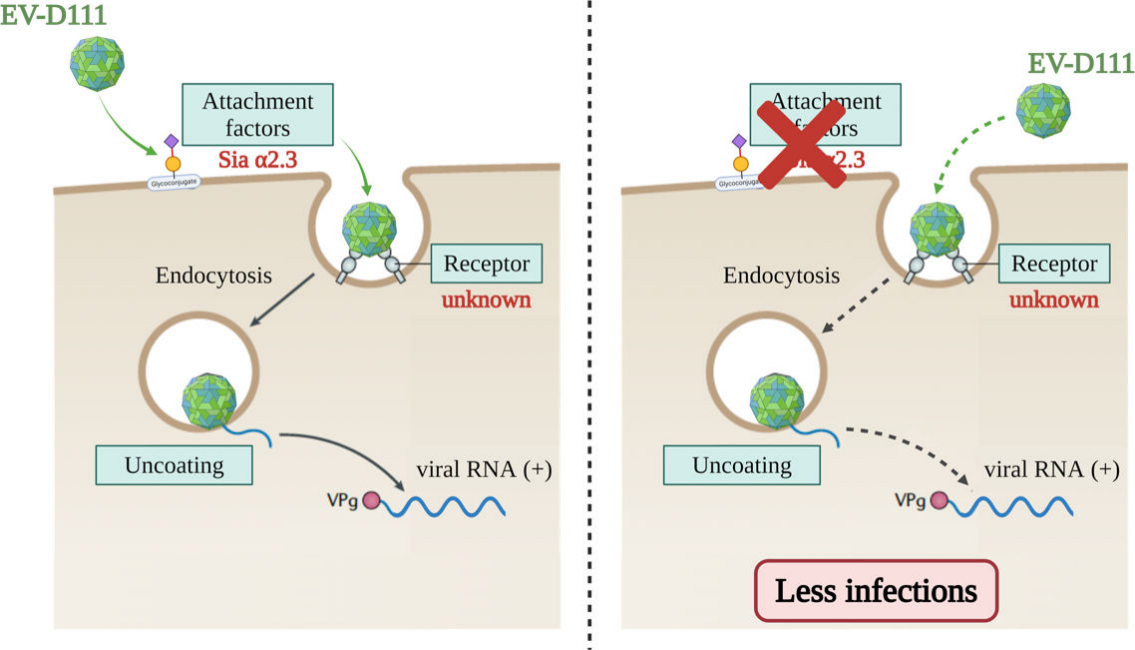
Potential entry mechanisms of EV-D111. The figure illustrates a model for the infectious entry of EV-D111. The virus could use a two-step mechanism for infection. First, EV-D111 binds to α2,3 Sia-bearing glycoproteins that concentrate infectious viral particles at the cell surface, which increases the probability of encountering the viral receptor. Interaction with this receptor triggers the endocytosis and conformational changes in the viral capsid, so the virion delivers its RNA genome across the endosomal membrane into the cytoplasm. This uncoating receptor is still unknown. In the absence of sialylated glycans, viral production is lower due to reduced entry into cells. The figure was created with BioRender.

In conclusion, our data reinforce the idea that the use of Sias is a trait shared by EV-Ds. We hypothesize that EV-D111 uses a two-step entry mechanism involving two different macromolecules, one allowing fixation and the other inducing decapsidation (Fig. 8). First, the virus binds Sias α2,3, which are at least attachment factors and enable its concentration on the cell surface and thus increasing the probability of encountering its receptor. Other glycosylated macromolecules with a Sia in their terminal position and other sugar derivatives, such as heparan sulfate or proteoglycans, could potentially be used by EV-D111 to bind cells, as is the case for EV-D94 (37). Once bound to its receptor, the virus will undergo endocytosis and a conformational change of the capsid, which leads to the opening of the viral particle and the release of the viral genome into the cytoplasm. Thus, the absence of Sias on the cell surface restricts EV-D111 infection without abolishing it, a hypothesis supported by our results. The viral receptor for EV-D111 is currently unknown, and in-depth studies are needed to identify it. However, we can assume that the EV-D111 entry receptor will be specific, as it is not using any of the known EV receptors (8, 10). A better understanding of EV-Ds biology is essential to understand its evolution and potential to emerge in humans.
